# Supplementary material for: Association between oral microbiome and seven types of cancers in East Asian population: a two-sample Mendelian randomization analysis
Source: Front Mol Biosci. 2023 Nov 21;10:1327893. doi: 10.3389/fmolb.2023.1327893 (PMC10702768; doi:10.3389/fmolb.2023.1327893)
Supplement: Supplementary file 1 [file DataSheet1.ZIP › Supplementary Materials/MR plots for tongue/tongue═╝/Lung cancer/pheno.3170_to_lung cancer_forest.pdf]

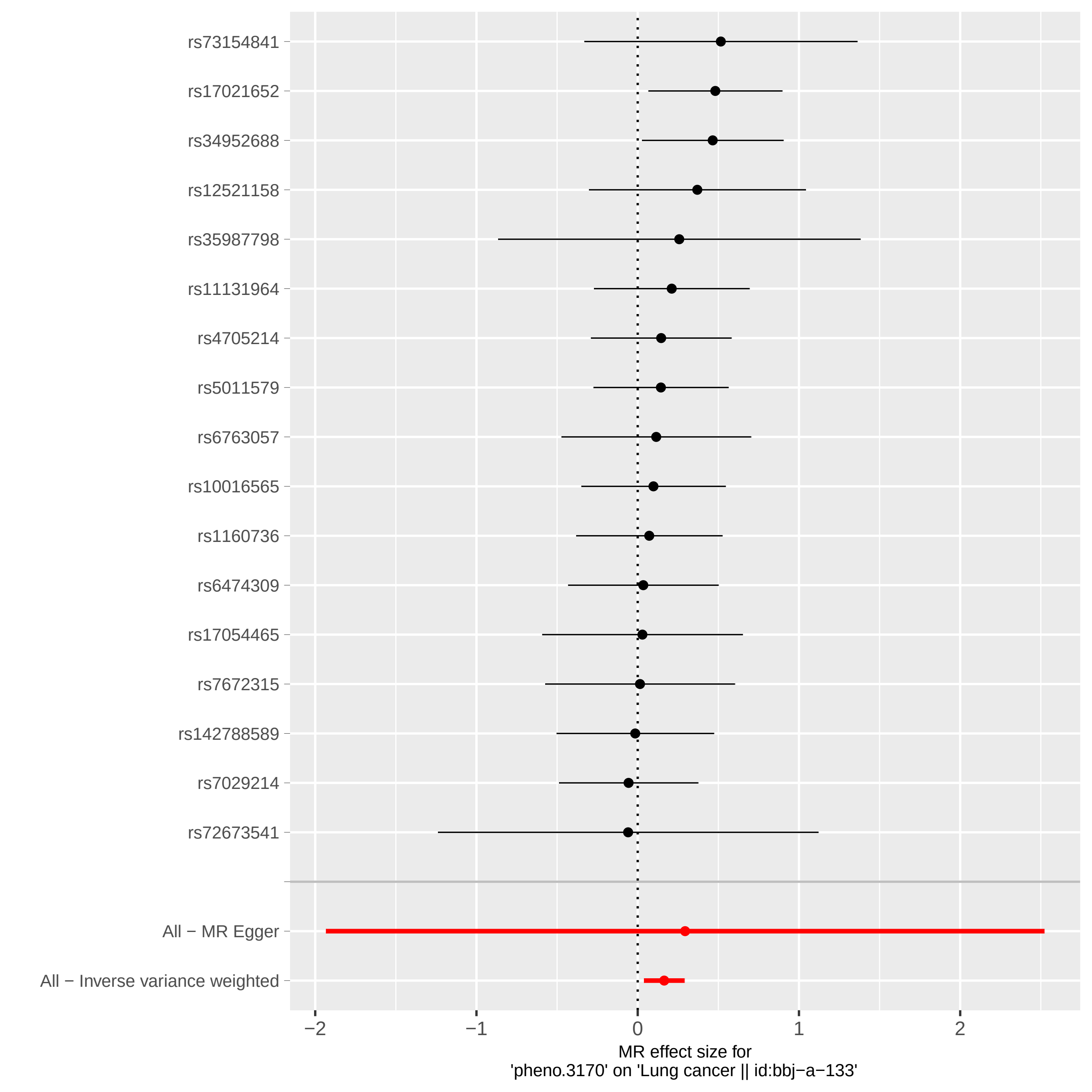

rs73154841
rs17021652
rs34952688
rs12521158
rs35987798
rs11131964
rs4705214
rs5011579
rs6763057
rs10016565
rs1160736
rs6474309
rs17054465
rs7672315
rs142788589
rs7029214
rs72673541
All − MR Egger
All − Inverse variance weighted
−2
−1
0
1
2
MR effect size for
'pheno.3170' on 'Lung cancer || id:bbj−a−133'
